# Medicinal plants used for the treatment of various skin disorders by a rural community in northern Maputaland, South Africa

**DOI:** 10.1186/1746-4269-9-51

**Published:** 2013-07-19

**Authors:** Helene De Wet, Sibongile Nciki, Sandy F van Vuuren

**Affiliations:** 1 Department of Botany, University of Zululand, Private Bag X1001, KwaDlangezwa 3886, South Africa; 2 Department of Pharmacy and Pharmacology, Faculty of Health Sciences, University of Witwatersrand, 7 York Road, Parktown 2193, South Africa

**Keywords:** Medicinal plants, Skin disorders, Lay people, Northern Maputaland, South Africa

## Abstract

**Background:**

Skin diseases have been of major concern recently due to their association with the Human Immunodeficiency Virus and Acquired Immunity Deficiency Syndrome (HIV/AIDS). The study area (northern Maputaland) has the highest HIV infection rate in South Africa, which made them more prone to a wide range of skin conditions. Fungal infections due to the hot climate and overcrowding households are common in this area, as well as burn accidents due to the use of wood as the major fuel for cooking. It is known that the lay people in this area depend on medicinal plants for their primary health care. However no survey has been done in northern Maputaland to document the medicinal plants used to treat various skin disorder.

**Methods:**

Interviews were undertaken at 80 homesteads, using structured questionnaires. The focus was on plants used for dermatological conditions and information regarding vernacular plant names, plant parts used, preparation (independently and in various combinations) and application was collected.

**Results:**

A total of 87 lay people, both male (22%) and female (78%) were interviewed on their knowledge of medicinal plants used to treat disorders of the skin. Forty-seven plant species from 35 families were recorded in the present survey for the treatment of 11 different skin disorders including abscesses, acne, burns, boils, incisions, ringworm, rashes, shingles, sores, wounds and warts. When searching the most frequently used scientific databases (ScienceDirect, Scopus and Pubmed), nine plant species (*Acacia burkei, Brachylaena discolor, Ozoroa engleri, Parinari capensis,* subsp*. capensis, Portulacaria afra, Sida pseudocordifolia, Solanum rigescens, Strychnos madagascariensis* and *Drimia delagoensis*) were found to be recorded for the first time globally as a treatment for skin disorders. Fourteen plant combinations were used. Surprisingly, the application of enema’s was frequently mentioned.

**Conclusions:**

The preference of traditional medicine over allopathic medicine by most of the interviewees strengthens previous studies on the importance that traditional medicine can have in the primary health care system in this rural community. Studies to validate the potential of these plants independently and in their various combinations is underway to provide insight into the anti-infective role of each plant.

## Background

Skin diseases occur worldwide and amount to approximately 34% of all occupational diseases encountered [1]. They affect people of all ages from neonates to the elderly and constitute one of the five reasons for medical consultation. Skin diseases have been of major concern recently due to their association with the Human Immunodeficiency Virus and Acquired Immunity Deficiency Syndrome (HIV/AIDS) [2]. Tschachler et al. [3] stated that more than 90% of HIV infected individuals develop skin and mucosal complications at some stage during the disease. Skin ailments present a major health burden in both developed and undeveloped countries. For example, in the United States, skin infections caused by methicillin-resistant *Staphylococcus aureus* (MRSA) result in approximately 126 000 hospitalizations while invasive MRSA results in approximately 94 360 infections and 18 650 deaths each year, a rate which exceeds that of AIDS [4]. According to the World Health Organization (WHO) [5], burns have also been a serious public health problem due to the global increase in burn mortality rates. In South Africa, over 19 500 fire-related deaths are reported annually and they rank among the 15 leading causes of death in children and young adults between the age of 5–29 years. Burn victims are also susceptible to serious and often fatal *Pseudomonas aeruginosa* infections [6]. Socio-economic environments such as household overcrowding play an enormous part in the spread of skin infections [7]. Furthermore, hot and humid climatic conditions exacerbate skin infections. These factors are particularly problematic in Sub-Saharan Africa where it was found that over 78 million people were infected with *Tinea capitis* (a superficial skin infection affecting the scalp) [8]. Although mortality rates for skin diseases are relatively low, they impact significantly on the quality of life and are often persistent and are difficult to treat.

Traditional medicinal resources, especially plants have been found to play a major role in managing skin disorders [1]. They have been employed in the treatment of skin ailments in many countries around the world where they contribute significantly in the primary health care of the population [1,4,9-13]. In South Africa, the majority of people especially in rural communities still depend, to a large extent, on medicinal plants to treat skin disorders [14-20]. This is not surprising, as South Africa is home to over 24, 000 higher plants species with approximately 3000 plant species been recorded by various cultural groups as part of their *materia medica*[21]. Furthermore, the use of medicinal plants to treat dermatological conditions is extensive. This was observed in a recent review whereby more than a hundred plant species were identified (from readily available literature) as being of importance when considering the traditional medicinal plant use in southern Africa to treat skin diseases [14]. Follow-up research on other traditional uses of medicinal plants, such as for the treatment of stomach ailments, sexually transmitted infections (STIs), respiratory complaints etc. have been given sufficient attention, but still lacking is attention given to the ethnobotanical plant use for skin diseases in remote geographical areas, such as that of northern Maputaland. Previously, two separate surveys on Khoi-San medicinal plant uses (Northern Cape Province and south eastern Karoo) recorded 18 plants species which are used to treat skin problems [15,22]. Another two surveys done in the Western Cape Province documented 57 plant species used by the “coloured” (mixed- race) people for the treatment of skin problems and the Bapedi traditional healers (Limpopo Province) used two exotic plants species to treat wounds [16,23,24]. Two ethnobotanical surveys done in the Eastern Province documented eight plant species for treating skin problems by the Xhosa people [17,20].

The aim of the present study was to collect ethno-medicinal knowledge from lay people in northern Maputaland for the application of medicinal plants as a treatment for skin disorders. This is the first survey done in this region which specifically focuses on dermatological ailments.

## Method

### Study area

**Figure 1:**
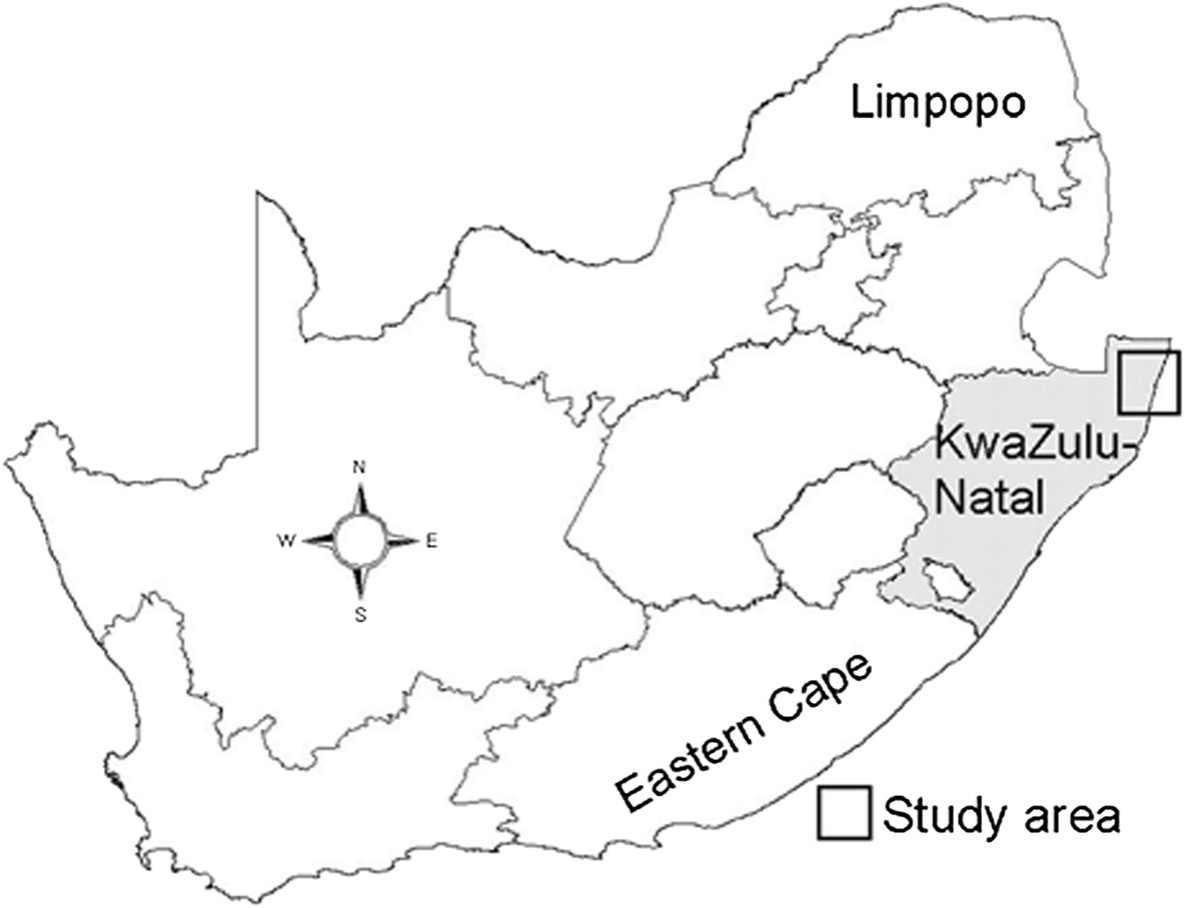
Study area – northern Maputaland located in KwaZulu-Natal province, South Africa.

The study area is located in the north eastern part of KwaZulu-Natal Province (Maputaland), South Africa (Figure 1). The area is situated between 32°22' and 32°52' latitudes and 27°15' and 27°30' longitudes and has a surface area of 3631 km^2^. The area experiences hot summers and comparatively mild winters with a mean annual temperature of 22°C. The hottest month is January with a mean temperature of 39.5°C. The region has dry winter months with a mean annual summer rainfall between 500 mm to 750 mm. The study area involved four regions (Mabibi, Mbazwana, Mseleni and Tshongwe) which primarily (99%) consists of informal settlements [25]. These regions belong to the Umhlabuyalingana local municipality, one of five municipalities that constitute the Umkhanyakude District. Each of the four selected regions is dominated by a different vegetation type, namely: the Maputaland Wooded Grassland (Mbazwana), Maputaland Coastal Belt (Mseleni), the Northern Coastal Forest (Mabibi) and the Tembe Bushveld Type (Tshongwe) [26]. The area consists of approximately 163 694 people and 27 006 households, which are predominantly isiZulu speaking people [25]. This area is regarded as one of the poorest regions in South Africa with 47% of the population having no formal income [25]. The region also lacks proper infrastructure such as electricity, clean water and sanitation facilities. Wood is the major fuel used for cooking (83.3%) followed by paraffin (7%) and gas (6%) [25].

### Ethnobotanical survey

The survey was carried out during June-July 2012. Ethical clearance (S378/08) was obtained from the University of Zululand’s Ethical Committee before the onset of the survey. A total of 80 homesteads were visited, 20 per region. The visited homesteads were identified by convenience sampling and people were interviewed using a structured questionnaire. Household residents were approached and the objective of the study was explained in isiZulu. A form of consent was signed prior to the interview. The questionnaire was designed to obtain information about the locality; socio-demographic details (age, gender and educational background); type (s) of skin disorders treated by the plants, vernacular names of plants mentioned, parts used, method of preparation, dosage forms and method of administration. The focus of the survey was to determine which plants that are growing in and around homesteads are used to treat skin disorders. Plant species recorded in the survey were collected and the voucher specimens have been deposited in the herbarium of the Department of Botany, University of Zululand, South Africa. Identities of plants sampled were authenticated by Dr THC Mostert from the University of Zululand and Mr M Ngwenya from the South African National Biodiversity Institute KwaZulu-Natal Herbarium.

## Results and discussion

### Socio-demographic information

A total of 87 lay people both male (22%) and female (78%) were interviewed on their knowledge of medicinal plants used to treat any disorders of the skin. Not many men are living at the homesteads as they are employed elsewhere in the country, thus explaining the predominantly female to male gender ratio interviewed. The interviewees’ age ranged between 14 and 88 years with the average age of 59. The sample for this survey is thus a reasonable represented age sample of the population in the study area, of which 53% is in the age bracket between 14–65 years (41% < 14 yrs and 6% > 65yrs) [25]. The level of education within the population is related to the prevailing poor socio-economic conditions with only 2% obtaining a tertiary education, 18% no formal education and 57% having not completed a grade 12 certificate (equivalent to a secondary school education) [25]. About 45% of the respondents in this survey have no formal education. The majority of the respondents (47%) claimed to obtain their ethnobotanical knowledge from their parents, 39% from their grandparents (of which some were traditional healers) and the remainder of the knowledge was obtained from neighbours, relatives, and friends. Irrespective of where the traditional knowledge came from, the important fact was that most of the lay people who had knowledge claimed to pass it on to the younger generation both orally and practically. Children observe the process of collecting and preparing the herbals. This corresponds with previous surveys done in the same area on the use of medicinal plants [27-29]. It seems that Zulu indigenous medicinal plant knowledge in rural areas is not declining as found in some other African countries like Ethiopia [30]. Although Cunningham [31] and Botha [32] stated that African medicinal plant practices are a highly specialised field, practiced only by herbalists and diviners, recent studies contradict this. More recently, it has been found that lay people not only practise local traditional medicinal treatment regimens, but also have good knowledge about medicinal plants [33-35]. This is true, especially for women in rural communities, who have had to cope daily with common diseases in the family.

### Plants mentioned to treat skin disorders

Forty-seven plant species were recorded in the present survey for the treatment of 11 different skin disorders (Additional file 1: Table S1). These disorders include abscesses, acne, burns, boils, incisions, ringworm, rashes, shingles, sores, wounds and warts. The 47 plant species belongs to 35 families, with Fabaceae (*sensu lato*) (eight species) being the most frequently represented family, followed by Asteraceae and Solanaceae (three species each), Anacardiaceae (two species) and the remainder, had one species each. Plant species from the family Fabaceae (*sensu lato*) are well known world-wide for the treatment of wounds [36-38]. The following nine plant species have been recorded for the first time globally for the use to treat skin disorders; *Acacia burkei*, *Brachylaena discolor*, *Ozoroa engleri*, *Parinari capensis*, subsp. *capensis*, *Portulacaria afra*, *Sida pseudocordifolia*, *Solanum rigescens*, *Strychnos madagascrariensis* and *Drimia delagoensis*. Although *A. burkei*, *B. discolor, O. engleri*, *P. capensis*, *P. afra*, *S. madagascariensis* and *D. delagoensis* have been recorded for other medicinal uses; *S. pseudocordifolia* and *S. rigescens* have no other documented medicinal uses. The indigenous herb *Senecio serratuloides* was by far the most frequently used species by the interviewees to treat skin disorders (17 interviewees). This is a well recorded medicinal plant in South Africa for the treatment of various skin disorders and wounds [21,29,39,40]. The second most mentioned species was the indigenous tree *Tabernaemontana elegans* (seven interviewees), followed by *Sclerocarya birrea* and *S. madagscariensis* (five interviewees), and *Dialium schlechteri* (four interviewees) (Additional file 1: Table S1). Hutchings et al. [18] has done an extensive inventory on Zulu medicinal plants and recorded uses for 1032 plant species, of which 190 species are used to treat various skin disorders. Only 19 plant species in the present survey correspond with Hutchings’s 190 plant species recorded. This low correlation of plant species is possibly because the research undertaken by Hutchings et al. [18] gained most of the information from literature and interviews with *nyangas* (Zulu herbal doctor’s), whereas this study focused on knowledge from the lay people. Furthermore, the remote areas in northern Maputaland (present study) were not the focus of Hutchings’s previous work [18]. This low correlation of plant use for skin diseases stresses the wealth of unrecorded indigenous knowledge still obtainable in rural communities, specifically in the Maputaland area.

**Figure 2:**
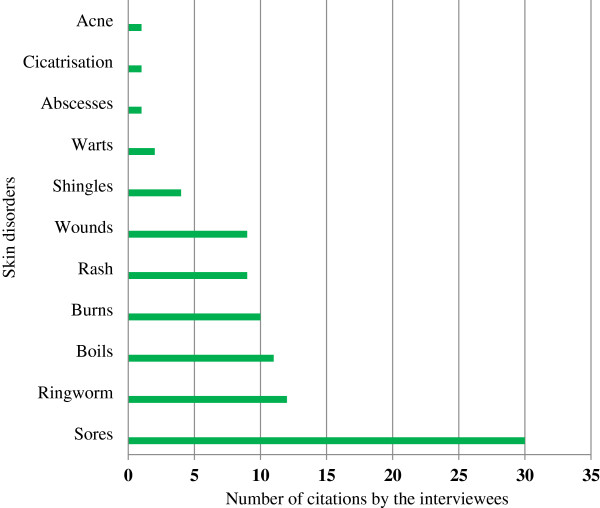
Frequency of different skin disorders treated by interviewees.

The present study is one of four surveys done in the same geographical area to document plants used by lay people for treating various infections. The first survey revealed 23 plant species which are used to treat diarrhoea [27], the second survey documented 33 plants species used for the treatment of respiratory infections [28] and the third survey documented 33 plant species for the treatment of STIs [29]. Three indigenous trees (*S. birrea*, *S. cordatum* and *T. sericea*) currently recorded in the present study are used by the lay people in this area to treat the symptoms of all three diseases (diarrhoea, STIs and respiratory infections), as well as being used for skin conditions. The pharmacological actions of these three trees have been previously investigated and various active compounds have shown antimicrobial and anti-inflammatory activities [21]. As sores were the most frequently treated skin condition (Figure 2), the antimicrobial and anti-inflammatory compounds may play a significant role in the healing process.

The plant species mentioned in Additional file 1: Table S1 were not only used independently, but also in various combinations. Fourteen plant species combinations were noted (Table 1). Different combinations were used, ranging from two plants in a combination (nine interviewees), five plants (two interviewees) and even six plants combined (two interviewees). Sores were mostly treated by these combinations. A survey done in India also revealed quite a few plant combinations for the treatment of wounds [41]. For example, a 12 plant combination is used externally to treat gangrene of wounds. In the present survey, *Senecio serratuloides* was used in four of the 14 plant species combinations. One of the vernacular names of *S. serratuloides* is “two day plant”, meaning it takes only two days for a wound or infection to be cured when this species is used [42]. This might explain its preferential use in combination. Furthermore, preference might be given to this species due to local availability as it is widely distributed in the study area.

**Table 1:** Plant combinations used to treat skin disorders by lay people in northern Maputaland

**Plant species use in combination**	**Parts used**	**Skin disorder treated**
*C. kirkii* + *S. serratuloides* + *R. multifidus* + *F. sur* + *E. elephantina* + *S. pirie* + *D. delagoensis*	Root + leaves + whole plant + bark + bulb	Sores
*A. burkei* + *O. engleri* + *S. birrea* + *S. cordatum* + *T. elegans* + *L. javanica*	Bark + leaves	Sores
*B. discolor* + *E. tirucalli* + *H. hemerocallidea* + *O. engleri* + *S. serratuloides*	Stem with leaves (twigs) + modified stem + corm + bark + leaves	Sores
*C. kirkii* + *E. elephantina* + *S. pirie* + *R. multifidus* + *D. delagoensis*	Roots + bark + whole plant + bulb	Shingles
*A. burkei* + *K. africana*	Bark	Ringworm
*B. pilosa* + *S. pseudocordifolia*	Leaves + whole plant	Ringworm
*C. inerme* + *D. cinerea*	Stem with leaves	Acne
*H. hemerocallidea* + *L. revoluta*	Corm + bulb	Ringworm
*H. hemerocallidea* + *S. rigescens*	Corm + fruit	Boils
*B. pinnata* + *S. serratuloides*	Leaves	Shingles
*S. birrea + S. cordatum*	Bark	Burns
*S. brachypetala* + *S. birrea*	Bark	Sores
*S. serratuloides* + *D. delagoensis*	Leaves + bulb	Rash
*S. madagascariensis + S. spinosa*	Leaves + fruit sap	Sores

Rural dwellers in the study area had showed comprehensive knowledge in the use of plant combinations to treat symptoms of different infections [27-29]. Using plants is a holistic approach to treat ailments and the synergy of plant combinations can have an even greater effect on treating infectious diseases. Some of the plants mentioned in this survey have shown many pharmacological properties which include antibacterial, antifungal, antiviral, anti-inflammatory, anti-oxidant, analgesic and astringent properties to mention a few [43-46]. These various different pharmacological actions of plants can validate the efficacy of poly-herbal usage by lay people to relieve a variety of symptoms (i.e. inflammation, infection, irritation etc.) associated with skin diseases.

### Plant parts used, preparation and administration methods

**Figure 3:**
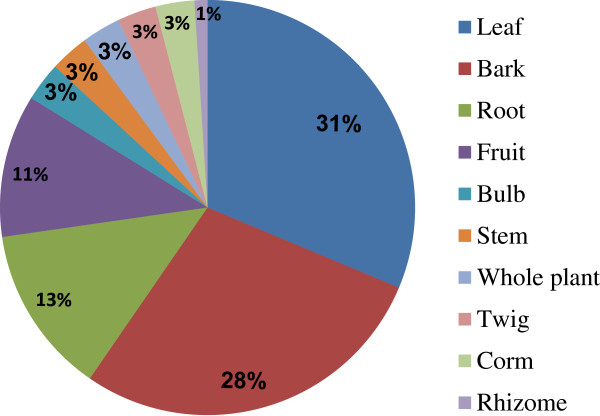
Percentage of different plant parts used to treat skin disorders by the rural people in Maputaland.

Figure 3 shows that leaves (31%) were the preferable plant part used to treat skin infections with bark a close second place (28%). In most other ethnobotanical studies where plants are used to treat various skin disorders, the leaves are also the preferable plant part used [11,13,14,37]. It was also noted in some cases, that different parts of the same plant species (either leaves or bark) could be used e.g. *S. madagascariensis* and *Z. mucronata* and bark or fruit in the case of *K africana* to treat ringworm. The recorded plant species were prepared in a variety of ways (Additional file 1: Table S1). The plant materials were used either fresh or dry in decoctions, macerations, pastes or powders. Administration of the different plant parts were mostly applied topically as a paste, powder, sap or latex on the affected skin area, followed by decoctions that were taken orally. Children’s dosages were usually less than those of adults. Baths were a popular way of treating rashes or itchy skin problems and steaming was used for acne. The most common treatments in other similar surveys are the use of decoctions as a wash, followed by application of pastes on affected areas [11,13,47]. In a recent review [14] on South African plants used for dermatological purposes, many of the applications (taken from the readily available ethnobotanical literature) were unspecified. Furthermore, the use of enemas has not been previously documented. In this survey, enemas are regularly used for the treatment of sores (Additional file 1: Table S1). The explanation given by the interviewees is that they believe the cause of the sores comes from inside the body and by inserting enemas; they are killing the root of the problem. Enemas are mostly taken simultaneously with decoctions for the same reason, to clean the body from the inside.

### Skin disorders treated by medicinal plants

The interviewees mentioned 11 different skin diseases which are treated with different plant-based medication (Figure 2). Sores (30 interviewees) are by far the most recorded skin problem treated; followed by ringworm infections (12 interviewees), boils (11 interviewees), burns (10 interviewees), wounds (nine interviewees) and rashes (nine interviewees). The skin diseases reported in the present study correspond with a similar study done by Njoroge and Bussmann [48] in Kenya, where sores are also the most frequently treated skin disease. The causes of these sores can be numerous. People with AIDS are easy susceptible to various skin disorders, bacterial infections (pyoderma) or tropical ulcers. These causes are common in developing countries, where living conditions are poor. Firstly, the HIV infection rate in the study area was estimated to be 15.21% of the population in 2007 [25]. This is 4.4% higher than the average South African HIV infection rate [25]. A recent study by De Wet et al. [29] in this area, also documented a high medicinal plant usage to treat various STIs, which probably can relate to the high treatment of sores in the present study. Secondly, high humidity, heat and lack of sanitation in this area are associated with an increased risk of fungal and bacterial skin infections. These conditions often exacerbate microbial infections of the skin, especially those caused by Group A *Streptococci* or *Staphylococcus aureus* infections [7]. Thirdly, is the frequency of particular infections associated with climate and socio-economic factors.

Tropical ulcers are mainly seen in Africa, India, and the western Pacific. These ulcers are usually caused by a combination of bacteria and/or unidentified spirochetes and are associated with poor living conditions and exposure to stagnant water and mud [7]. Ringworm can also be expected, because of climatic conditions and overcrowding. It is mostly caused by *Tinea capitis* a common contagious fungal infection which can spread extensively in homes and schools. In many parts of Africa more than 30% of children in primary schools are affected [7,48]. A recent survey done in the north eastern state of India revealed that 46 plant species are used exclusively to treat various dermatophytes, including ringworm [9]. None of the plant species mentioned in this study for the treatment of ringworm correspond with the ones used in India, although the two surveys shared the genus *Solanum*. Two other common skin treatments are burns and wounds which are likely to happen in rural communities where wood, paraffin, gas and candles are mostly used as fuel for cooking (83.3%) and light [25]. A study done on burn cases treated at the Ngwelezana Hospital Burns Unit in northern KwaZulu-Natal, shows that children under the age of 12 made up 69.5% of all admissions [49]. Most of their burns were caused by hot water and food. Direct flame burns accounted for 19.6% of the patients treated [49]. Most of the recorded wound treatments in the present study were for new wounds, inflicted during wood collecting and cultivation of the fields (home-gardens). The remedies were mostly used to stop the flow of blood, cleansing of the wounds and as dressings for burns and wounds. Various other studies globally have also reported plant remedies for the treatment of wounds [10,11,36,41,50]. Another skin disease mentioned in the present survey was shingles, which is rarely mentioned in other ethnobotanical surveys. Possibly, shingles caused by the *Herpes zoster* virus (characterized by a painful skin rash with blisters) is not known by rural communities and may be more often classified as rashes or sores. Shingles and other rashes are very common in immuno-compromised individuals with HIV/AIDS, which as previously mentioned, is very high in the study area.

### The use of traditional medicine versus western medicine

Although 91.6% of the population in the study area have access to health facilities (two hospitals and 17 clinics) [25], the majority of the interviewees preferred the use of medicinal plants for the treatment of skin disorders. Various explanations were given (medicinal plants are effective, cheap, readily available and used for cultural reasons), some of which are similar to those previously mentioned in surveys done in this region [27-29]. Apart from these reasons, interviewees believed that some of the skin diseases such as acne, warts and shingles cannot be treated with western medicine. Some interviewees who suffered from acne and warts were apparently not healed by western practitioners, but after using traditional medicinal plants e.g. *C. inerme*, *D. cinerea*, *S. incanum* and *S. spinosa* (Additional file 1: Table S1), they were seemingly cured. Some western practitioners in this area recommended the use of traditional medicine as a first line of treatment and should this prove ineffective, proceed to clinics or hospitals. Twenty nine of the interviewees claimed to use both traditional and western medicine in combination. This tendency also occurs in other cultures (Chinese, Indian etc.) where traditional and western medicine occurs side by side in a complementary way [51-53]. The former is being used to treat self-terminating or chronic conditions and the latter to treat more serious and acute conditions [54]. Ten of the interviewees do not use any traditional medicine, mostly because of religious beliefs. This was also reported in a previous survey in this region [28]. Another reason for not always using traditional medicine was the negative associations with taste.

## Conclusions

The rural people of Maputaland, South Africa make use of at least 50 remedies deriving from 47 plant species for the treatment of skin and soft tissue disorders, clearly indicating the importance the local people have for medicinal plants. *Senecio serratuloides* was the most frequently used plant species by the rural dwellers to treat general sores and burns. The curative properties of *S. serratuloides* are well known in the Zulu *materia medica*, which explains its high usage. The contribution of new ethnobotanical knowledge is reflected by the fact that nine plant species were documented for the first time to treat skin diseases (*A. burkei*, *B. discolor*, *O. engleri*, *P. capensis*, subsp. *capensis*, *P. afra*. *S. pseudocordifolia*, *S. rigescens*, *S. madagascariensis* and *D. delagoensis*). Leaves were the preferable plant part used and were mostly applied topically as a paste, powder or sap on the affected skin area. This is encouraging for sustainable development purposes as the traditional use favours plant parts that can be regrown with ease. It is also noted that lay people are using their medicinal plants conservatively as it is a valuable free source in their primary health care system. None of the plant species are threatened or endangered with respect to their conservation status, except *H. hemerocallidea* which is reported to be declining in the wild [55]. Further studies are under way to validate the possible antimicrobial efficacies of these plants (independently or their use in various combinations) against skin relevant pathogens. New knowledge to this effect may offer superior efficacies of plant species other than *H. hemerocallidea.* This information can be communicated back to the lay people with recommendations of substitutive uses which will protect the declining *H. hemerocallidea* populations.

The preference of traditional medicine over allopathic medicine by most of the interviewees strengthens previous documentation on the importance that traditional medicine can have in the primary health care system in this rural community. The lay people who use traditional medicine may not understand the scientific rational behind their medicines, but there is no doubt that they perceive from personal experience, that some of these medicinal plants are highly effective.

## Competing interests

The authors declare that they have no competing interest.

## Authors’ contributions

HDW and SVV conceptualized the study and wrote the manuscript, SN and HDW carried out the field work. All authors read and approved the final manuscript.

## Supplementary Material

Additional file 1: Table S1 Medicinal plants used for the treatment of skin disorders in a rural community in northern Maputaland [11,18,19,21,35,36,39-42,56-85]. Click here for file
