# Supplementary material for: Medicinal plants used for the treatment of various skin disorders by a rural community in northern Maputaland, South Africa
Source: J Ethnobiol Ethnomed. 2013 Jul 19;9:51. doi: 10.1186/1746-4269-9-51 (PMC3724715; doi:10.1186/1746-4269-9-51)
Supplement: Additional file 1: Table S1 — Medicinal plants used for the treatment of skin disorders in a rural community in northern Maputaland [11,18,19,21,35,36,39-42,56-85]. [file 1746-4269-9-51-S1.doc]

**Table 1: Medicinal plants used for the treatment of skin disorders in a rural community in northern Maputaland**

| **Botanical name**  **(voucher no)** | **Family** | **Common name** | **Plant part(s)**  **used** | **Number of times quoted** | **Methods of preparation and administration** | **Other reported uses for skin disorders** |
| --- | --- | --- | --- | --- | --- | --- |
| *Acacia burkei* Benth.  (S.Nciki 25) | Fabaceae (*sensu lato*) | Umkhaya | Bark | 2 | (a) Mix a handful of chopped bark with the same amount of bark of *Ozoroa engleri*, *Sclerocarya birrea*, *Syzygium cordatum*, *Tabernaemontana elegans* and two handfuls of fresh *Lippia javanica* leaves in 2 L of water and boil for 2 hrs. Strain when cool and half a glass (±125 mL) is taken by adults and a size eight syringe (±240 mL) as an enema for the treatment of sores. Children take quarter a glass (±62 mL) of the decoction and use a size two syringe (±60 mL) as an enema. The decoction is taken three times a day for two weeks after which the sores begin to heal.  (b) Macerate half a handful of fresh chopped bark with a handful of *Kigelia africana* bark in 1 L of water. Infants take 1 tbsp (tablespoon) three times a day for a week to treat ringworm which appears as red spots at the back of their necks. | None found |
| *Albizia adianthifolia* (Schumach.) W. Wight  (S.Nciki 36) | Fabaceae (*senso latu*) | Igowane | Bark | 3 | (a) Two handfuls of fresh pounded bark are mixed in 2 L of warm water. One cup of maceration is added in bathing water once a day for a week to treat scaly, itching and burning body rash.  (b) Two handfuls of fresh pounded bark are mixed in 2 L of cold water in which the affected person is bath once a day for two weeks (approximate duration of rash).  (c) The same amount of bark as in (b) is mixed with 10 L of warm water and 1 L of this mixture is used as an emetic once a day for 10 days to treat the rash. | Scabies [39]; eczema [18]; skin diseases [21] |
| *Albizia versicolor* Welw. ex Oliv. (S.Nciki 5) | Fabaceae (*senso latu*) | Umphezi | Bark or root | 1 | A handful of fresh bark or 30 cm of fresh root is chopped and boiled in 1L of water for five minutes. The strained cooled down decoction is used as an enema (size eight syringe) once a day for two days for the treatment of sores. | Skin rashes [39]; wounds, sores [19] |
| *Annona senegalensis* Pers. (S.Nciki 8) | Annonaceae | Umphfwa | Root | 1 | A handful of chopped fresh roots are boiled in 1 L of water for 20 min. Half a glass of the cooled strained decoction is taken with soft porridge once a day for the treatment of body sores. | Leprosy [56]; wounds, dermatosis [57]; abscesses and wounds [18; 58]; skin rashes [59] |
| *Bidens pilosa* L.  (S.Nciki 19) | Asteraceae | Uqadolo | Leaves | 2 | (a) Mix a handful of the fresh leaves with a handful of *Sida pseudocordifolia* whole plant and apply as a paste onto the ringworm infection after an incision is made. Applied once a day for a week.  (b) The sap of a handful of fresh leaves is used as an astringent to new wounds. | Astringents to cuts, wounds [40, 60, 61, 62]; burns [63; 64]; fungal skin infection [18]; skin rashes [65] |
| *Brachylaena discolor* DC. (S.Nciki 6) | Asteraceae | Iphahla | Twigs (leaves and stems) | 1 | Mix a handful of chopped fresh twigs with a handful each of *Euphorbia tirucalli* stem, leaves of *Ozoroa engleri*, *Senecio serratuloides* and one chopped corm of *Hypoxis hemerocallidea* in10 L of water and boil for 3 hrs. One tbsp of the decoction is taken by adults three times a day for a week for body sores. Children take 1 teaspoon (±5 mL tsp) of the decoction. | None found. |
| *Canthium inerme* (L.f) Kuntze  (S.Nciki 3) | Rubiaceae | Umvuthwamini | Twigs | 1 | One fresh twig is chopped with that of *Dichrostachys cinerea* and bring to boil in 5 L of water. Steam twice a day for a week to treat acne. | Sores [39] |
| *Cladostemon kirkii* (Oliv.) Pax & Gilg  (Nzama 13) | Capparaceae | Isidumbu | Roots | 3 | (a) Two handfuls of chopped fresh roots are macerated in 2 L of water. Adults take 3 tbsp three times a day to treat body sores. Children take 1 tsp.  (b) Roots in combination with roots of *Elephantorrhiza* *elephantina*, *Sarcophyte pirie* and bark of *Ficus sur*, leaves of *Senecio serratuloides* and a chopped bulb of *Drimia delagoensis* are boiled in 1 L of water for 5 min. Half a glass of the strained decoction is taken by adults three times a day for the treatment of sores. Children take 1 tbsp of the decoction.  (c) Chopped root in combination with 30 cm fresh or dry chopped root of *Elephantorrhiza elephantina*, *Sarcophyte pirie* as well as a chopped bulb of *Drimia delagoensis* and one handful of *Ranunculus multifidus* (whole plant) are boiled in 2 L of water. Half a glass of the strained decoction is taken by adults once a day for the treatment of shingles. Children take 1 tsp full of the medication. | Boils [18] |
| *Dalbergia obovata* E. Mey. (S.Nciki 9) | Fabaceae (*senso latu*) | Isijwandlube | Leaves | 1 | A handful of crushed leaves is made into a paste and applied to burns once a day for two days after which the burned area become dry and starts to heal. | Mouth sores in infants [39] |
| *Dialium schlechteri* Harms (S.Nciki 31) | Fabaceae (*senso latu*) | Umthiba | Bark | 4 | (a) A handful of dry or fresh bark is pounded into a powder or paste which is applied directly onto the burn area. It is applied once and not removed until the burned area becomes dry.  (b) A handful of fresh or dried bark is pounded into a powder. A tsp of powder is mixed with oil or petroleum jelly and applied to a new wound once a day for two weeks.  (c) Half a handful of dry bark is pounded into a powder which is applied onto a cut boil to reduce inflammation. | Burns [18] |
| *Dichrostachys cinerea* (L.) Wight & Arn.  (S.Nciki 10) | Fabaceae (*senso latu*) | Ishashane | Twigs | 1 | It is used in combination with *Canthium inerme* as described at *C. inerme* to treat acne. | Wounds [11, 21, 39]; scabies and sores [19]; abscesses [42]; variety of skin diseases [65] |
| *Elephantorrhiza elephantina* (Burch.) Skeels (S.Nciki 12) | Fabaceae (*senso latu*) | Umsehle | Root | 2 | (a) It is used in combination with *C. kirkii, S. pirie,* *D. delagoensis and R. multifidus* as described at *C. kirkii* to treat shingles.  (b) It is use in combination with *C. kirkii*, *F. sur*, *S. pirie*, *S. serratuloides*, *R. multifidus* and *D. delagoensis* as described at *C. kirkii* to treat sores. | Wounds [39]; acne [21, 66]; dermatitis [67] |
| *Euphorbia tirucalli* L. (T.York 37) | Euphorbiaceae | Umduze | Modified stem | 3 | It is used in combination with *B. discolor*,  *H. hemerocallidea*, *O. engleri* and *S. serratuloides* as described at *B. discolor* to treat body sores. | Warts [68] |
| *Ficus sur* Forssk*.*  (S.Nciki 2) | Moraceae | Umkhiwane | Bark | 1 | It is used in combination with *C. kirkii, E.* *elephantina*,  *R. multifidus,* *S.pirie*, *S. serratuloides* and  *D. delagoensis* as described at *C. kirkii* to treat body sores. | Burns and leprosy [18]; skin rashes [42]; boils, wounds [36] |
| *Garcinia livingstonei* T. Anderson (S.Nciki 21) | Clusiaceae | Umphimbi | Bark | 1 | A handful of bark is pounded into a paste that is directly applied to burns once a day for a week, after which the burned area becomes dry and heals. | Skin rashes [69] |
| *Hewittia malabarica* L. (Suresh) (S.Nciki 14) | Convolvulaceae | Inhlanzandulo | Leaves | 1 | A handful of crushed leaves are incorporated into a paste and applied onto cut boils and abscesses once a day for a week. | Wounds [70] |
| *Hibiscus surattensis* L. (S.Nciki 28) | Malvaceae | Icishamlilo | Root | 2 | (a) A handful of root is pounded into a paste that is directly applied to burns once a day for a week.  (b) Fresh roots are boiled in 1 L of water for 30 min. Two tbsp. of the decoction is taken by adults for the treatment of sores. Children take one tsp three times a day for a month. | Cuts [71]; abscesses, boils, wounds [62] |
| *Hypoxis hemerocallidea* Fisch., C.A. Mey. & Avé-Lall. (Nzama 38) | Hypoxidaceae | Ilabatheka, Inkomfe | Corm | 3 | (a) Two corms are chopped together with two corms of *Ledebouria obovata* and boiled in 2 L of water for 30 min. Two tbsp. of the decoction is taken by children three times a day for the treatment of ringworm.  (b) One corm is chopped in combination with the fruits of *Solanum rigescens* and boiled in 5 L of water. One tbsp. of strained decoction is taken three times a day for less than a month by adults for the treatment of boils. Children take one tsp of the decoction.  (c) The corm is used in combination with *B. discolor*,  *E.* *tirucalli*, *O. engleri* and *S. serratuloides* as mentioned at *B. discolor* to treat body sores. | Burns [18] |
| *Bryophyllum pinnata* (Lam.) Oken (S.Nciki 40) (= *Kalanchoe pinnata* Lam.) | Crassulaceae | Umvuthuza | Leaves | 1 | A handful of leaves in combination with a handful of dried or fresh leaves of *S. serratuloides* are mixed with brake-fluid (hydraulic fluid) and Jik (bleach) and pasted over shingles twice a day for three days. | Abscesses and boils [13, 8]; abscesses, burns, wounds [72]; scabies [73]; new wounds [74] |
| *Kigelia africana* (Lam.) Benth. (S.Nciki 27) | Bignoniaceae | Umvongothi | Bark and fruit | 3 | (a) Half a handful of bark or one dry fruit is incinerated and the ash is applied with oil onto ringworm infection after an incision is made.  (b) Two handful of bark are chopped and boiled in 1L of water for 1 hr. Half a glass of the decoction is taken three times a day for a month for cicatrisation of new surgical incisions.  (c) It is used in combination with *A. burkei* as previously described at *A. burkei* for the treatment of ringworm. | Acne, boils, eczema, dermatitis, skin fungal infections, leprosy, skin cancer, ulcers, wounds [5; 54;75;76;77] |
| *Ledebouria revoluta* (L.f.) Jessop (S.Nciki 13) | Hyacinthaceae | Umhlahlane, Inhlwathiyesikhotha | Bulb | 1 | It is used in combination with *H. hemerocallidea* as described at *H. hemerocallidea* for the treatment of ringworm. | Skin irritations, sores, wounds [18] |
| *Lippia javanica* (Burm. F.) Spreng.  (S.Nciki 26) | Verbenaceae | Umsuzwane | Leaves | 1 | It is used in combination with *A. burkei*, *O. engleri*, *S*. *cordatum*, *S. birrea* and *T. elegans* as described at *A. burkei* for the treatment of sores. | Measles, urticarial and febrile rash [39] acne [78] |
| *Momordica balsamina* L*.* (S.Nciki 11) | Curcubitaceae | Umkaka | Leaves | 2 | (a) Take two handfuls of leaves and macerated it in 2 L of warm or cold water. The maceration is used to bath in for the treatment of body rash and half a cup can be used for rubbing in to the infected body part.  (b) Half a handful of fresh leaves are made into a paste and applied onto ringworm infections on the scalp after an incision is made. This is done once a day for two days. | Seeds are used for frost bite and burns [39]; fruits are used for wounds [78], scabies, skin allergies [18, 71] |
| *Opuntia stricta* (Haw.) Haw. (Nzama 41) | Cactaceae | Umdolofiya | Modified stem | 1 | Latex of the cut cladode is dropped onto the newly cut opened boil, once a day for two days in order to reduce inflammation. |  |
| *Ozoroa engleri* R.Fern. & A.Fern (S.Nciki 37) | Anacardiaceae | Isifice | Bark | 2 | (a) It is used in combination with *A. burkei*, *L. javanica*, *S*. *cordatum*, *S. birrea* and *T. elegans* as described at *A. burkei* for the treatment of sores.  (b) It is used in combination with *B. discolor*, *E. tirucalli*, *H. hemerocallidea*, *O. engleri* and *S. serratuloides* as described at *B. discolor* to treat sores. | None found. |
| *Parinari capensis* Harv*.* subsp*. capensis*  (S.Nciki 7) | Chrysobalanaceae | Amabulwa | Rhizome | 1 | One fresh rhizome is chopped and boiled in 1 L of water for less than 30 min. Adults take half a glass of strained decoction in combination with soft porridge to treat body sores. | None found. |
| *Portulacaria afra* Jacq.  (S.Nciki 4) | Portulacaceae | Ufenisi | Leaves | 2 | Two handfuls of leaves are macerated in 2 L cold water. The prepared maceration is used to bath in for the treatment of rash and chronic sores. | None found. |
| *Ranunculus multifidus* Forssk. (S.Nciki 23) | Ranunculaceae | Ixhaphozi | Whole plant | 3 | (a) Five fresh entire plants are grounded and rolled into five balls and allowed to dry, after which it is mixed with 2 L of cold water. One tbsp. of the maceration is taken three times a day for the treatment of shingles. Half a cup is used as an enema by adults. Dosage for children is a size two syringe.  (b) It is used in combination with *C. kirkii*, *E. elephantina*, *S. pirie* and *D. delagoensis* as described at *C. kirkii* to treat shingles.  (c) It is used in combination with *C. kirkii*, *E. elephantine*, *F. sur*, *S. pirie*, *S. serratuloides* and *D. delagoensis* as described at *C. kirkii* to treat sores. | Open sores [18]; wounds [40] |
| *Sarcophyte pirie* Hutch. (Nzama 29) (= *S. sanguinea* Sparm) | Balanophoraceae | Umavumbuka | Bark | 2 | (a) It is used in combination with *C. kirkii*, *E. elephantine*, *F. sur*, *R. multifidus*, *S. serratuloides* and *D. delagoensis* as described at *C. kirkii* to treat sores.  (b) It is used in combination with *C. kirkii*, *E. elephantine*, *F. sur*, *R. multifidus* and *D. delagoensis* as described at *C. kirkii* to treat shingles. | Acne and other skin eruptions [79] |
| *Schotia brachypetala* Sond. (S.Nciki 22) | Fabaceae (*senso latu*) | Umgxamu | Bark | 1 | A handful of fresh bark in combination with a handful of  *S*. *birrea* bark is macerated in warm water for less than 2 hrs. The strained maceration is used by adults as an enema (size eight syringe) three times a day for the treatment of sores. Children use a size two syringe. | Acne [35, 18] |
| *Sclerocarya birrea (A. Rich.)* Hotsch. (S.Nciki 17) | Anacardiaceae | Umganu | Bark | 5 | (a) Dried pounded bark in combination with *S. cordatum* bark is applied to burns, twice a day for two weeks after which burned area becomes dry and starts to heal.  (b) A pinch of pounded dry bark is applied onto a newly cut boil to reduce inflammation.  (c) It is used in combination with *A. burkei*, *L. javanica*, *O. engleri*, *S. cordatum* and *T. elegans* as described at *A. burkei* to treat sores.  (d) It is used in combination with *S. brachypetala* as described at *S. brachypetala* to treat sores. | Skin eruptions [68]; insect bites, burns, abscesses, spider bites [80] |
| *Senecio serratuloides* DC. (S.Nciki 1) | Asteraceae | Unsukumbili | Leaves | 17 | (a) Ash of incinerated leaves is mixed with oil or petroleum jelly and applied as a paste on burns.  (b) Ash of incinerated leaves is applied to open sores, wounds, cuts and abrasions.  (c) A handful fresh or dried leaves is boiled in 1 L of water for 15 min. Half a glass of the decoction is taken by adults three times a day for the treatment of rashes. Children take 1 tbsp. The decoction can also be taken as an enema. Adults use a size eight syringe and children a size two. Alternatively the concoction can be used to bathe in.  (d) Powdered dry leaves mixed with oil can be pasted on sores once a day for two days. The paste is also applied onto ringworm infections after an incision is made.  (e) Fresh leaves are mix with 5 L of warm water in combination with one chopped bulb of *D. delagoensis*. The prepared maceration is used for bathing twice a day for two weeks for the treatment of rashes.  (f) It is used in combination with *C. kirkii*, *E. elephantina*, *F. sur*, *R. multifidus*, *S. pirie* and *D. delagoensis* as described at *C. kirkii* to treat sores. | Burns, sores [39, 40; 42]; septic sores [18] |
| *Sida pseudocordifolia* Hochr. (S.Nciki 20) | Malvaceae | Umphipho | Whole plant | 1 | It is used in combination with *B. pilosa* as described at *B. pilosa* to treat ringworm. | None found. |
| *Solanum incanum L.* (S.Nciki 38) (*= S. panduriforme* E. Mey.) | Solanaceae | Intuma | Fruit | 1 | The content of a fresh fruit is rubbed onto a wart once a day for a week to reduce inflammation after which it will flake off. | Mouth sores [81]; itching blisters [39]; wounds [19]; burns [18]; skin ailments [40] |
| *Solanum rigescens* Jacq. (S.Nciki 39) | Solanaceae | Umthuma | Fruit | 2 | (a) Six fruits are boiled in 4 L of water until they burst. Half a cup of the strained decoction is taken by adults three times a day for less than a month to treat boils. Children take 1 tbsp.  (b) It is used in combination with *H. hemerocallidea* as described at *H. hemerocallidea* to treat the outbreak of boils. | None found. |
| *Strychnos madagascariensis* Poir  (S.Nciki 35) | Strychnaceae | Umkwakwa | Leaves and Bark | 5 | (a) A handful of leaves are mixed with the sap from the fruit of *S. spinosa* to form a paste which is applied to sores on the body twice a day for a week.  (b) A handful of pounded bark is applied to open sores.  (c) A handful of chopped bark is boiled in one cup of water for 10 min. Half a cup is used twice a day to wash ringworm infections after an incision is made.  (d) A handful of dry bark is pounded and the powder is applied to burns once a day for two weeks. | None found. |
| *Strychnos spinosa* Lam.  (S.Nciki 34) | Strychnaceae | Umhlala | Fruit | 2 | (a) Unripe fruit is cut open and the contents are rubbed over the surface of warts once a day for less than a month.  (b) It is used in combination with *S. madagascariensis* as described at *S. madagascariensis* to treat sores. | Scabies [18]; wounds [36] |
| *Syzygium cordatum* Hochst. ex C. Krauss. (S.Nciki 18) | Myrtaceae | Umdoni | Bark | 3 | (a) A handful of dry or fresh chopped bark is boiled in 2 L of water. Half a glass of the decoction is taken by adults twice a day for the treatment of sores. Children take one tbsp. | Wounds [18]; mouth sores [35]; skin rashes [5] |
|  |  |  |  |  | (b) It is used in combination with *A. burkei*, *L. javancia*, *O. engleri*, *S. birrea* and *T. elegans* as described at *A. burkei* to treat sores.  (c) It is used in combination with *S. birrea* as described at *S. birrea* to treat burns. |  |
| *Tabernaemontana elegans* Stapf  (S.Nciki 24) | Apocynaceae | Umkhahlu | Leaf or fruit and bark | 7 | (a) Latex of leaves is applied directly to new wounds to arrest bleeding and promote healing, two or three times a day for three days.  (b) Leaf or fruit latex is applied onto ringworm infections after an incision is made once a day for a week.  (c) Bark is used in combination with *A. burkei*, *L. javanica*, *O. engleri*, *S. birrea* and *S. cordatum* as mentioned at *A. burkei* to treat sores. | Wounds [35] |
| *Terminalia sericea* Busch ex. DC  (S.Nciki 16) | Combretaceae | Ikonono | Leaves | 1 | A handful of leaves are crushed into a paste and applied directly onto burns once a day for less than a week. The same amount of leaves can be mixed with one cup of cold water and used to wash the burned area. | Wounds [18]; anal eczema and measles [82] |
| *Drimia delagoensis* (Baker) Jessop (S.Nciki 15) (= *Urginea delagoensis* Baker) | Hyacinthaceae | Isuzi, Umahlanganisa | Bulb | 3 | (a) The bulb is used in combination with *C. kirkii*, *E. elephantina*, *F. sur*, *R. multifidus*, *S. pirie* and *S*. *serratuloides* as described at *C. kirkii* for the treatment of sores.  (b) The bulb is use in combination with *C. kirkii*, *E. elephantina*, *R. multifidus* and *S. pirie* as described at *C. kirkii* for the treatment of shingles  (c) The bulb is use in combination with *S. serratuloides* as described at *S. serratuloides* to treat body rash. | None found. |
| *Waltheria indica* L.  (S.Nciki 29) | Malvaceae | Isinama | Root | 2 | (a) Debarked roots are pounded into a paste and applied on burns once a day for two weeks.  (b) Fresh pounded roots are applied as an astringent to new wounds once a day for three days. | Wounds [8; 36, 39]; sores [68]; boils [36] |
| *Withania somnifera* (L.) Dun. (S.Nciki 15) | Solanaceae | Ubuvhimba | Root | 1 | Dried root is incinerated and the ash is mixed with oil or Vaseline and applied to shingles, twice a day for two weeks. Alternatively grounded roots are boiled in 1 L of water and 1 tbsp. of the decoction is taken by adults three times a day for two weeks. | Ringworm [39]; abscesses, cuts, wounds [17, 66]; carbuncles [8] |
| *Ximenia caffra* Sond.  (S.Nciki 30) | Olacaceae | Umthunduluka | Twigs | 1 | One or two fresh twigs are chopped and boiled in 2 L of water, which are taken by adults twice a day for two weeks for the treatment of sores. Children take 1 tbsp. | Open wounds [18]; dermatitis [83]; abscesses [82] |
| *Xysmalobium undulatum* (L.) Aiton f. (Hlatshwayo 11) | Apocynaceae | Ishongwe | Root | 1 | Dried pounded root is applied as an astringent to new wounds, until the wound becomes dry. | Acne, skin eruptions, wounds, sores [39]; wounds [21, 66] |
| *Zanthoxylum capense* (Thunb.) Harv. (S.Nciki 33) | Rutaceae | Umnungwane | Root | 1 | A fresh grounded root is boiled in a cup of water and half a cup is used to wash new open wounds, twice a day for a week. | Boils and acne (18; 19]; sores [84]; shingles [35] |
| *Ziziphus mucronata* Willd. (S.Nciki 32) | Rhamnaceae | Umlahlankosi | Leaves and roots | 3 | (a) Half a handful of crushed leaves are placed on to a newly cut boil in order to reduce inflammation. The same amount of dried leaves is grounded and the powder is placed on the boil for the same purpose once a day for two days.  (b) A handful of pounded roots is placed on a newly cut boil and then wrapped with a bandage. | Boils, sores [39]; abscesses [56]; wounds [85] |
